# Editorial: Bioleaching and biorecovery of critical raw materials from secondary sources

**DOI:** 10.3389/fmicb.2024.1395820

**Published:** 2024-04-10

**Authors:** Laura Castro, Ruiyong Zhang, Jesús Angel Muñoz, Wolfgang Sand

**Affiliations:** ^1^Department of Chemical and Materials Engineering, Complutense University of Madrid, Madrid, Spain; ^2^CAS Key Laboratory of Marine Environmental Corrosion and Bio-fouling, Institute of Oceanology, Chinese Academy of Sciences, Qingdao, China; ^3^Guangxi Key Laboratory of Marine Environmental Science, Institute of Marine Corrosion Protection, Guangxi Academy of Sciences, Nanning, China; ^4^Aquatic Biotechnology, University of Duisburg-Essen, Essen, Germany; ^5^Institute of Biosciences, Freiberg University of Mining and Technology, Freiberg, Germany

**Keywords:** bioleaching, biorecovery, critical raw materials, secondary sources, metal recycling

Bioleaching has emerged as a sustainable and green technology for metal recovery and is an alternative to conventional extractive processes with a reduced effect on climate change (Mohan and Joseph, 2020). This approach has been used commercially for the extraction of metals from low-grade ores and attracts attention for applying innovative methods to mobilize critical metals from secondary sources (Castro et al., 2020; Danouche et al., 2024), such as e-wastes (Baniasadi et al., 2019; Adetunji et al., 2023), spent catalysts (Moosakazemi et al., 2023), fly ash, red mud (Hedrich and Schippers, 2021), and scraps (Ilyas et al., 2013), to contribute to the development of a circular economy.

There is a growing interest in mine tailings as well as several end-of-life products to start value recovery from waste, with a focus on strategic metals (Zhang et al., 2020; Schueler et al., 2024). Bioleaching makes use of microorganisms' metabolic activities, which produce lixiviants, such as organic acids and siderophores that facilitate the dissolution of metals in the presence of large amounts of organic compounds.

Moreover, biorecovery involves the use of microorganisms or biomasses to selectively accumulate and concentrate target metals from leachates. The integration of biohydrometallurgical techniques into the recycling loop has great potential to promote a circular economy where valuable metals are efficiently extracted, recycled, and reused. In this way, the dependency on primary resources may be reduced, and the ecological footprint of the mining industry may be mitigated.

The main purpose of this Research Topic is to present recent advances in the use of bioleaching and biorecovery for critical raw materials from industrial wastes. Within this topic, four articles have been published on innovative bioleaching processes, including the design and optimization of bioreactors and the utilization and characterization of valuable sources of strategic metals.

Red mud is a hazardous alkaline waste from the digestion of bauxite in the Bayer process for alumina production, which could be treated economically for its richness in critical metals. Zhang et al. studied both, the dealkalinization and the solubilization of metals (i.e., Al, Ce, and Y), from red mud through sulfur (S^0^) oxidation as catalyzed by the moderately thermophilic bacterium *Sulfobacillus thermosulfidooxidans*. The bioleaching process is mediated by biogenic H_2_SO_4_. The optimal conditions depend on the S^0^:red mud mass ratio and aeration to promote S^0^ bio-oxidation. The authors suggest the reduction of liquid waste by media recirculation or the use of by-products from gas and/or coal desulfurization processes as S^0^ sources for the bacteria in large-scale industrial applications to reduce the operational costs.

Li et al. investigated a cost-effective alternative for uranium extraction from complex ores. Their research studies the feasibility of sulfur enhancement for uranium bioleaching in column reactors using a designed mixed culture of *Acidithiobacillus ferrooxidans, Acidithiobacillus thiooxidans*, and *Leptospirillum ferriphilum* from a refractory uranium ore. The sulfur enhancement favored the uranium solubilization, which caused a final recovery of 86.2%. The uranium bioleaching kinetics are fitted with internal diffusion through a product layer-controlled model. The sulfur enhancement could strengthen the porosity of the passivation layer and improve the ore permeability. The bacterial community also played a key role, and a controlled balance of the iron-oxidizing bacteria and sulfur-oxidizing bacteria increased the uranium bioleaching process.

Gallium is used in integrated circuits and advanced electronic components because it allows low energy consumption and high computation speed. However, minerals containing gallium are scarce in nature. Nowadays, the demand for this element is high, which is leading to the development of effective recovery processes from secondary resources such as mine tailings or electronic recycling material. Bioleaching was considered an extractive method by Chung et al. The authors obtained genomic sequences of five heterotrophic bacteria, isolated from different Portuguese mines, to identify potential mechanisms involved in bioleaching ability and strategies to overcome metal(loid) toxicity. The presence of effective resistance mechanisms to gallium and arsenic was particularly important for bioleaching Ga from GaAs and GaN.

Spiess et al. have for the first time studied the recovery of Zn from bioleachate using a microbial electrolysis cell. The proposed approach combines bioleaching and bioelectrochemistry to develop a fully biotechnological metal recovery process. During Zn recovery from bioleachate, aluminum precipitation and iron reduction were observed. The energy consumption for Zn recovery using a microbial electrolysis cell was notably lower than electrowinning. After 3 months of operation, the electroactive species *Enterococcus* was enriched on the anode from sewage sludge.

To sum up, biohydrometallurgy is a promising approach to extracting critical metals from non-conventional sources. In this way, it can contribute to a circular economy. However, more detailed studies are necessary to evaluate the optimization of bioleaching processes for different wastes, scaling up operations for industrial applications, and addressing microbial resistances to target metals. Overcoming these challenges in future is crucial for the successful integration of biohydrometallurgical techniques into recycling processes.

## Author contributions

LC: Writing – original draft, Writing – review & editing. RZ: Writing – original draft, Writing – review & editing. JM: Writing – original draft, Writing – review & editing. WS: Writing – original draft, Writing – review & editing.
